# PCGF1 promotes epigenetic activation of stemness markers and colorectal cancer stem cell enrichment

**DOI:** 10.1038/s41419-021-03914-2

**Published:** 2021-06-19

**Authors:** Guangyu Ji, Wenjuan Zhou, Jingyi Du, Juan Zhou, Dong Wu, Man Zhao, Liping Yang, Aijun Hao

**Affiliations:** Key Laboratory for Experimental Teratology of Ministry of Education, Shandong Key Laboratory of Mental Disorders, Department of Anatomy and Histoembryology, School of Basic Medical Sciences, Cheeloo College of Medicine, Shandong University, Jinan, Shandong 250012 China

**Keywords:** Cancer stem cells, Colorectal cancer

## Abstract

Colorectal cancer (CRC) stem cells are resistant to cancer therapy and are therefore responsible for tumour progression after conventional therapy fails. However, the molecular mechanisms underlying the maintenance of stemness are poorly understood. In this study, we identified PCGF1 as a crucial epigenetic regulator that sustains the stem cell-like phenotype of CRC. PCGF1 expression was increased in CRC and was significantly correlated with cancer progression and poor prognosis in CRC patients. PCGF1 knockdown inhibited CRC stem cell proliferation and CRC stem cell enrichment. Importantly, PCGF1 silencing impaired tumour growth in vivo. Mechanistically, PCGF1 bound to the promoters of CRC stem cell markers and activated their transcription by increasing the H3K4 histone trimethylation (H3K4me3) marks and decreasing the H3K27 histone trimethylation (H3K27me3) marks on their promoters by increasing expression of the H3K4me3 methyltransferase KMT2A and the H3K27me3 demethylase KDM6A. Our findings suggest that PCGF1 is a potential therapeutic target for CRC treatment.

## Introduction

Colorectal cancer (CRC) is one of the most common malignant tumours of the digestive system with high morbidity and mortality rates. CRC is the world’s fourth most deadly cancer, with almost 900,000 deaths annually [1, 2]. More than 50% of patients who undergo radical surgery and standard chemoradiotherapy will eventually experience metastasis and relapse [3]. Thus, it is of great importance to identify key regulators that control CRC risk stratification to aid in the development of more effective therapeutic drugs.

Numerous studies have confirmed that there is a small group of cells in tumours with great self-renewal and multidirectional differentiation abilities. This group of cells is called cancer stem cells (CSCs) [4]. CSCs have been associated with the initiation of CRC for a long time. Increasing evidence has highlighted the role of cancer CSCs in CRC aggressiveness, metastasis, chemoresistance and subsequent tumour recurrence [5, 6]. Therefore, understanding the self-renewal and differentiation mechanisms of CSCs has important clinical significance [7]. Proteins have been proposed as colorectal cancer stem cell markers, including CD133, CD44, ALDH1A1, LGR5 and several others, and these proteins are considered prognostic indicators of CRC [8]. A better understanding of the mechanisms underlying the regulation of colorectal cancer stem cell markers could provide novel therapeutic strategies specifically targeting CSCs.

PCGF1, also named nervous system polycomb 1 (NSPc1), belongs to the polycomb group (PcG) protein family, which includes the PRC1 and PRC2 complexes [9]; among them, PRC1 is divided into classical PRC1 and non-classical PRC1 based on different of PCGF proteins [10–13]. The PcG protein family plays an important role in development and stem cell maintenance [14], and their dysregulation has been closely linked to oncogenesis and cancer stem cell phenotypes [15, 16]. PCGF1 functions as a key epigenetic regulator of embryonic stem cell self-renewal and early embryogenesis [17–20] and is abundantly expressed in several cancers [21, 22]. PCGF1 expression is increased in oral squamous cell carcinoma stem cells [23] and promotes glioma stem cell self-renewal by downregulating the expression of RDH16 [22]. The above consequences of PCGF1 misregulation have been linked to CSCs, which can produce tumours through a combination of increased self-renewal and the lack of complete cellular differentiation [10]. However, the role of PCGF1 in CRC remains largely unknown. In this study, we found that PCGF1 was highly expressed in CRC and inversely associated with the prognosis of CRC patients. Downregulation of PCGF1 inhibited the proliferation and enrichment of colorectal cancer stem cells. Mechanistically, PCGF1 enhanced the expression of colorectal cancer stem cell markers and affected the histone methylation modification of CSC markers. Thus, our findings revealed that PCGF1 might be a promising therapeutic target for CRC.

## Materials and methods

### Cell culture and sphere formation

Human colorectal cancer cell lines (HCT116, LoVo, SW480, SW620 and DLD-1) and the human normal colonic epithelial cell line HCoEpiC used in this study were obtained from American Type Culture Collection (ATCC) (Manassas, VA, USA). All the cell lines were cultured in Dulbecco’s modified Eagle’s medium (DMEM) (HyClone) containing 10% foetal bovine serum (FBS; ExCell Bio) and 1% penicillin-streptomycin-amphotericin B (MacGene) at 37 °C in a humidified incubator with 5% CO_2_. The cell lines were authenticated by short tandem repeat (STR) profiling and tested free of mycoplasma.

For the sphere formation assay, 500 HCT116 and SW620 cells were digested with 0.25% trypsin (Gibco, USA), washed with PBS and subsequently cultured in serum-free DMEM/F-12 medium (Gibco, USA) containing 2% B27 (Gibco, USA), 20 ng/ml EGF (Invitrogen, USA), and 20 ng/ml bFGF (R&D, USA) in 6-well ultra-low attachment culture plates for several days. Then, the diameter of the spheres ≥ 50 μm in each well was counted under a microscope.

### Cell counting Kit-8 (CCK-8) assay

Cell proliferation was determined using Cell Counting Kit-8 (CCK-8; APExBIO Technology LLC) assays. Briefly, 8 × 10^3^ cells/well were seeded in 96-well plates. Subsequently, cell proliferation was detected with CCK-8. The absorbance of each well was measured using a microplate reader at a wavelength of 450 nm.

### Flow cytometry (FCM) analysis

The apoptosis rate was evaluated using an Annexin V-FITC (AV)-Propidium Iodide (PI) (AV-PI) Staining Apoptosis Detection Kit (C1062; Beyotime Institute of Biotechnology) according to the manufacturer’s instructions. The number of apoptotic cells was analysed using a CytoFLEX S flow cytometer (Beckman Coulter, Inc.).

### RNA extraction and reverse transcription-quantitative PCR (RT-qPCR)

Total RNA was extracted using TRIzol reagent (TransGen Biotech Co., Ltd.). cDNA was synthesized using a RevertAid™ First Strand cDNA Synthesis Kit (Thermo Fisher Scientific). Then, cDNAs were amplified using reverse transcription-quantitative (RT-qPCR). qPCR was performed in triplicate with the following thermocycling conditions: 95 °C for 10 min, followed by 40 cycles at 95 °C for 15 s and 60 °C for 60 s on a CFX96™ instrument (Real-Time System; Bio-Rad Laboratories, Inc.). Cycle thresholds were normalized to that of the internal control β-actin. The relative expression of mRNAs was quantified using the 2^−ΔΔCq^ method. The sequences of the primers are shown in Table 1.

**Table 1:** Primer sequences.

Gene	Forward primer	Reverse primer
β-Actin	TGGCACCACACCTTCTACAA	CCAGAGGCGTACAGGGATAG
PCGF1	GCCGGCTACTTCGTGGAT	GGTCCAGTTTGAGGTTGAGC
CD133	TGAACTGAGGCAGCTTCCACCC	CGACAGTCGTGGTTTGGCGTT
CD44	TTCATAGAAGGGCACGTGGT	CATTGGGCAGGTCTGTGAC
ALDH1A1	TGGAGTCAATGAATGGTGGAA	TGATTTGGCCACATACACCA
Oct4	GTCGCTCTCATCTTGCTCAATTCC	GGGATTGAGACATGCAGGAAGTG
EZH2	AATCAGAGTACATGCGACTGAGA	GCTGTATCCTTCGCTGTTTCC
KDM6A	TTCCTCGGAAGGTGCTATTCA	GAGGCTGGTTGCAGGATTCA
KDM6B	CGCTGCCTCACCCATATCC	ATCCGCGACCTCTGAACTCT
KMT2A	ACTCAGTCTGGGGAATCTGC	TTGTCCTTCTCCACGCTCTT
KMT2B	CCTTGGGACTCGAATCAGGT	GCAGCTTTGCCTCTTCCTTT
KMT2C	CCCTTTCCAGAGAGCCAGAA	CCAACAGAGACCAGGGAGTT
CD133 #1 (ChIP)	CCAGAAGCCGGGTCATAAATAAT	AGCGAACCCGTCCACTCCTCACT
CD133 #2 (ChIP)	GAACTGCGGGGAGAGCGTGGTG	TCCCCGAGAGCGAGTCCGAAGTC
CD44 (ChIP)	ATCTAGGTGTTCTAGCTCCTGAATC	CTACCATTCCTAGAGAAGGGAGTC
ALDH1A1 (ChIP)	CTTGGTCCCTAGGTTCTCACAA	CACTTAAGCAGATCTTTTCTGCC

### Western blot analysis

Colorectal cancer stem cells were lysed in RIPA buffer (Beyotime Biotechnology, Shanghai, China) with protease inhibitors. The lysates were separated on SDS-polyacrylamide gels and transferred to 0.22 μm PVDF membranes. The membranes were incubated with 5% skim milk for 2 h at RT and incubated with the primary antibodies at 4 °C overnight. Primary antibodies against the following molecules were used at the following dilutions: PCGF1 (1:1000; ab183499, Abcam), CD133 (1:1000; ab19898, Abcam), SOX2 (1:1000; ab97959, Abcam), Oct4 (1:1000; ab18976, Abcam), cleaved PARP1 (1:1000; ab32064, Abcam), H3K4me3 (1:1000; CST#9751, CST), H3K27me3 (1:1000; CST#9733, CST), H3K9me3 (1:1000; CST#13969, CST), H3K9/K14ac (1:1000; CST#9677, CST), H3K18ac (1:1000; CST#9675P, CST), H3 (1:1000; CST#4499, CST), and β-actin (1:1000; HC201, TransGen Biotech). The membranes were incubated with HRP-conjugated goat anti-mouse (1:5000; HS201-01, TransGen Biotech) or goat anti-rabbit (1:5000; HS101-01, TransGen Biotech) secondary antibodies for 1 h at room temperature after washing. Protein expression was detected with Immobilon™ Western Chemiluminescent HRP Substrate (Millipore). The protein bands were analysed using ImageJ software (64-bit).

### Plasmid construction, lentivirus packaging and infection

In brief, full-length PCGF1 sequences were cloned into a PLVX-EGFP vector, and shRNAs targeting human PCGF1 were cloned into the pLKO-shRNA vector. To obtain lentiviruses, PLVX-PCGF1 or pLKO-PCGF1 shRNA plasmids were cotransfected with PAX and PMD into 293T packaging cells using PEI (Solarbio, China). The supernatant (containing virus) was harvested at 48 and 72 h after filtering with a 0.22 μm filter membrane. All primer sequences are listed in Table 2.

**Table 2:** Oligonucleotides used for plasmid construction.

*Plasmid constructs*	
PCGF1	F: CCGCTCGAGATGGCGTCTCCTCAGG
	R: CGCGGATCCACCCTCCTCTTCTCTTTCACAC
*Knockdown shRNA*	
PCGF1 sh#1	F: CCGGGCAAGACAGTGAAGAGAAACGCTCGAGCGTTTCTCTTCACTGTCTTGCTTTTT
	R: AATTAAAAAGCAAGACAGTGAAGAGAAACGCTCGAGCGTTTCTCTTCACTGTCTTGC
PCGF1 sh#2	F: CCGGGCAGCTTTGACCACTCTAAAGCTCGAGCTTTAGAGTGGTCAAAGCTGCTTTTT
	R: AATTAAAAAGCAGCTTTGACCACTCTAAAGCTCGAGCTTTAGAGTGGTCAAAGCTGC
PCGF1 sh#3	F: CCGGGCCACTGCTCAACCTCAAACTCTCGAGAGTTTGAGGTTGAGCAGTGGCTTTTT
	R: AATTAAAAAGCCACTGCTCAACCTCAAACTCTCGAGAGTTTGAGGTTGAGCAGTGGC
PCGF1 sh#4	F: CCGGGCTCCAGTCAGTGTACAAGATCTCGAGATCTTGTACACTGACTGGAGCTTTTT
	R: AATTAAAAAGCTCCAGTCAGTGTACAAGATCTCGAGATCTTGTACACTGACTGGAGC

*F* forward primer, *R* reverse primer.

For lentivirus infection, CRC cells were dissociated into single cells and seeded in 6-well plates (1 × 10^6^ cells/well). Eight hours later, the cells were incubated with 2 ml virus solution supplemented with 200 μl FBS, 200 μl DMEM and 10 μl polybrene (10 mg/ml, H8761, Solarbio). Four days later, the effect of the virus on expression was evaluated at the mRNA and protein levels. Stably expressing cells were established by puromycin (10 μg/ml, P8230, Solarbio) selection.

### Haematoxylin and eosin (H&E) staining

The tumour sections were cut to a thickness of 4 μm using a microtome. The sections were dewaxed using xylene and soaked in ethanol with gradient concentrations of ethanol for 5 min. After being washed with distilled water for 5 min, Haematoxylin (C0107; Beyotime Institute of Biotechnology) was added dropwise to stain the tissue for 15 min. The stained tissue was disposed of in 1% hydrochloric acid ethanol (Merck, Germany) for colour separation for 10 s. The 0.25% eosin dye (C0109; Beyotime Institute of Biotechnology) solution was added for counterstaining for 5 min, and tissues were dehydrated by soaking in gradient concentration alcohol for 5 min. All these steps were performed at room temperature.

### Immunofluorescence (IF) staining

Immunofluorescence staining was performed as described previously [24]. CSCs were incubated on poly-L-lysine-coated coverslips and cultured for several days. Then, the cells on the coverslips were fixed with 4% paraformaldehyde for 30 min. The coverslips were washed with PBS, followed by permeabilization with 0.3% Triton X-100 for 30 min at RT. The coverslips were blocked with 10% goat serum and incubated with anti-CD133 (1:200; AF0090, Beyotime), anti-LGR5^+^ (1:200; ab75732, Abcam), anti-Ki-67 (1:200; ab16667; Abcam) and anti-KDM6A (1:200; A8159, ABclonal) antibodies. Subsequently, the cells were incubated with fluorescence-conjugated secondary antibodies (Abbkine-488 and Abbkine-594 (Abbkine)) for 1 h. The nuclei were stained with DAPI (Sigma-Aldrich Corp., St. Louis, MO, USA). The stained cells were observed with a fluorescence microscope (Olympus, Japan).

### Immunohistochemical (IHC) analysis

The mouse tumour was removed and then fixed with 10% formaldehyde. After ethanol dehydration and paraffin embedding, the tumour was cut into sections with a thickness of ~0.4 μm. Then, the sections were dewaxed, rehydrated and immersed in 3% hydrogen peroxide for 10 min. After washing with PBS, the sections were incubated with PCGF1 (bs-5734R, Bioss) overnight at 4 °C. The sections were incubated with the secondary antibody for 60 min at RT the next day. Finally, DAB was added and incubated for 30 s.

### Chromatin immunoprecipitation (ChIP)

Chromatin immunoprecipitation (ChIP) was performed as described previously [24]. It was conducted using a SimpleChIP^TM^ Enzymatic Chromatin IP Kit (Magnetic Beads) (CST, #9003). CSCs were treated with 1% formaldehyde for 15 min at RT, which served as a cross-linking agent, and were then incubated with glycine at RT for 10 min to terminate the cross-linking reaction. Then, CSCs were sonicated to shear the DNA into chromatin fragments of 200–500 bp. The supernatants were incubated overnight with anti-H3K27me3 and anti-H3K4me3 antibodies or a control antibody (anti-IgG). Then, the supernatants were subjected to the washing, elution and cross-link reversal processes that were performed according to the manufacturer’s instructions. The purified DNA fragments were subjected to real-time PCR. The primer sequences are listed in Table 1.

### Tumour xenografts

The female BALB/c nude mice (4–6 weeks) used in this study were purchased from GemPharmatech Co., Ltd., which is fully accredited by the Institutional Animal Care and Use Committee. All animals were housed in cages in a 12/12 h light/dark cycle at a temperature of 24 °C and humidity of 50–70% and allowed free access to food and water. The sample size is not predetermined by statistical method but rather based on preliminary experiments. Nude mice were randomly divided into the control and experimental groups. The phenotype was analysed by a blind investigator. Mice were injected subcutaneously in the right flank with a serial number of viable negative control and stable knockdown of PCGF1 HCT116 cells (shPCGF1) (5 × 10^4^, 5 × 10^6^) in 100 μl of PBS. The tumours were measured and histologically confirmed. Two perpendicular tumour dimensions (*a* = length, *b* = width) were measured with Vernier callipers and used to calculate the volume (*V*; mm^3^) according to the formula *V* = (*a* × *b*^2^)/2. All experiments were approved by the Animal Care Committee of Shandong University.

### GEPIA and GEDS dataset analysis

The Gene Expression Profiling Interactive Analysis (GEPIA) database (http://gepia.cancer-pku.cn/index.html) was used to analyse the RNA sequencing expression data from 8587 normal and 9736 tumour tissue samples from the TCGA and GTEx projects [25]. GEPIA provides customizable functions, including tumour/normal differential expression analysis, profiling according to cancer types or pathological stages, patient survival analysis, similar gene detection, correlation analysis and dimensionality reduction analysis.

The Gene Expression Display Server (GEDS) database (http://bioinfo.life.hust.edu.cn/web/GEDS/) curated and normalized the gene expression data at the mRNA, miRNA and protein levels in 23,315, 9009 and 9244 samples, respectively, from 40 tissues (The Cancer Genome Atlas (TCGA) and Genotype-Tissue Expression (GETx)) and 1594 cell lines (Cancer Cell Line Encyclopedia (CCLE) and MD Anderson Cell Lines Project (MCLP)) [26].

### Statistical analyses

GraphPad Prism version 8 (GraphPad Software, Inc.) was used for statistical analysis. The samples which met proper experimental conditions were included in the analysis. Comparisons between two groups were analysed using two-tailed Student’s *t*-test. Comparisons among multiple groups were analysed using one-way ANOVA followed by Dunnett’s post hoc test. Data are presented as the mean ± SEM. *P* < 0.05 was considered to indicate a statistically significant difference.

## Results

### PCGF1 is upregulated in colorectal cancer

**Fig. 1:**
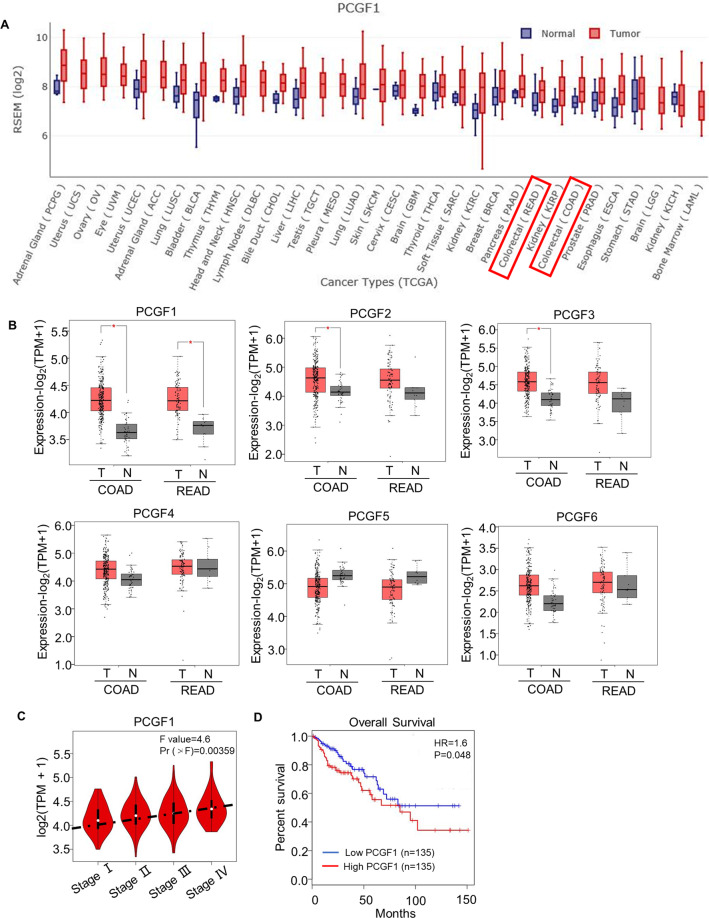
PCGF1 is upregulated in colorectal cancer. **A** Expression of PCGF1 in different types of tumours in the GEDS database. The red boxes refer to colorectal READ and COAD. **B** Expression of PCGF1-6 in colorectal cancer in the GEPIA database. COAD (num(T) = 275; num(N) = 41), READ (num(T) = 92; num(N) = 10). T, tumour; N, normal. **C** PCGF1 expression levels in specimens with various clinical stages. **D** Kaplan–Meier analyses of the OS rate in CRC patients in the GEPIA database with high or low expression of PCGF1 (*n* = 135, log-rank test). The hazard ratio (HR) and *P* value (log-rank test) for each comparison are shown.

We first analysed the expression of PCGF1-6 in various types of tumours using the Gene Expression Display Server (GEDS) database and found that PCGF1 in tumour tissues is generally elevated compared with that in normal controls, including colorectal READ (rectum adenocarcinoma) and COAD (colon adenocarcinoma) (Fig. 1A and Supplementary Fig. 1). We also analysed PCGF1-6 expression in publicly available human colorectal cancer datasets of the Gene Expression Profiling Interactive Analysis (GEPIA) database and found that PCGF1 in colorectal cancer tissues was significantly upregulated compared with that in normal control tissues (*P* < 0.05). In contrast, PCGF2, PCGF3, PCGF4, PCGF5 and PCGF6 did not show significantly different expression between tumour and normal colorectal tissues (Fig. 1B). Subsequent analysis revealed that PCGF1 expression was higher in advanced malignant tumours and significantly associated with the clinical stage (stages I–IV) (Fig. 1C). Importantly, the GEPIA database and log-rank tests revealed that PCGF1 mRNA levels greater than the mean were associated with decreased overall survival (OS) rates (HR = 1.6; *P* = 0.048) (Fig. 1D).

### PCGF1 is highly expressed in colorectal cancer stem cells

**Fig. 2:**
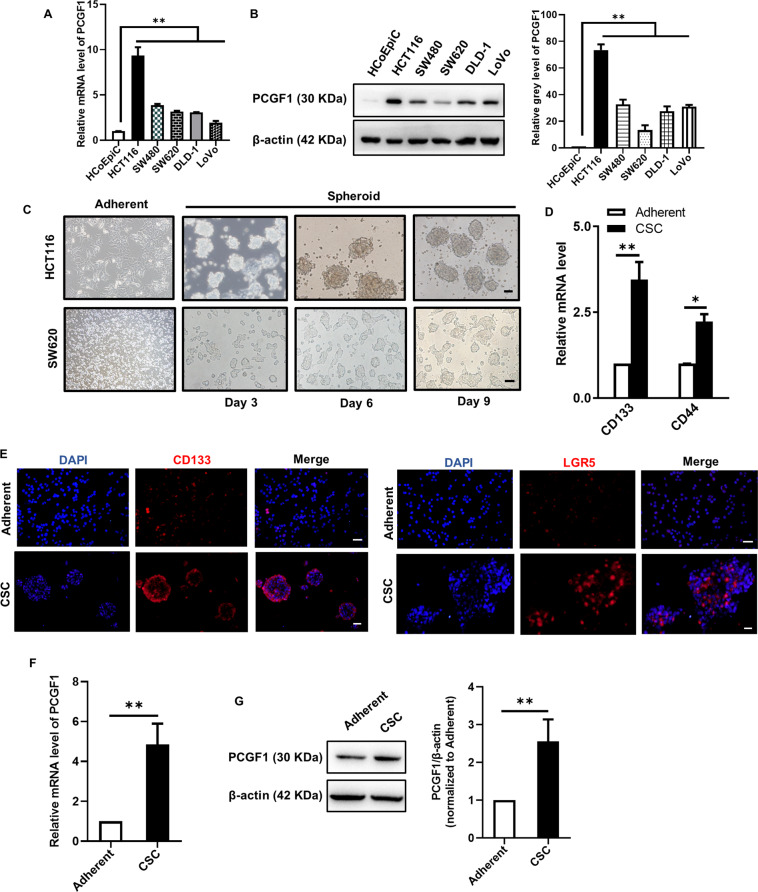
PCGF1 is highly expressed in colorectal cancer stem cells. **A**, **B** RT-qPCR and western blot analysis of the PCGF1 mRNA and protein levels in HCoEpiCs and a panel of CRC samples, and the relative intensity values (PCGF1/β-actin) were measured with ImageJ software. **C** HCT116 and SW620 cells were cultured in a serum-free environment for 9 days. Sphere morphology was photographed using light microscopy. Scale bar, 50 µm. **D** RT-qPCR analysis of the expression of CD133 and CD44 in colorectal cancer stem-like cells and adherent cells. **E** The expression levels of the stem cell markers CD133 and LGR5 were detected by immunofluorescence staining. Scale bar, 50 µm. **F**, **G** RT-qPCR and western blot analysis of PCGF1 mRNA and protein levels in adherent and CSCs, and the relative intensity values (PCGF1/β-actin) were measured with ImageJ software. **P < 0.01, *P < 0.05, one-way ANOVA and *t*-test. Data are shown as the mean ± SEM of at least three replicates. β-actin was used as a loading control.

To verify the results predicted by database analysis, we compared the mRNA and protein expression of PCGF1 in HCT116, SW480, SW620, DLD-1 and LoVo CRCs with that in HCoEpiCs (normal intestinal epithelium cells) and found that PCGF1 was more highly expressed in CRCs than in HCoEpiCs (Fig. 2A, B). To further explore the relationship between PCGF1 expression and tumour malignancy, we used sphere cultures to induce spheroid body formation in HCT116 colorectal cancer cells to enrich CSCs (Fig. 2C). The stem cell-related properties of the spheroids and the adherent cells were further examined via the stemness-related markers CD133, CD44 and LGR5 through RT-qPCR and immunofluorescence staining (Fig. 2D, E). PCGF1 mRNA and protein were more highly expressed in CSCs than in bulk CRCs (Fig. 2F, G). All the results demonstrate that PCGF1 may be associated with the progression and malignancy of colorectal cancer.

### PCGF1 promotes colorectal cancer stem cell enrichment

**Fig. 3:**
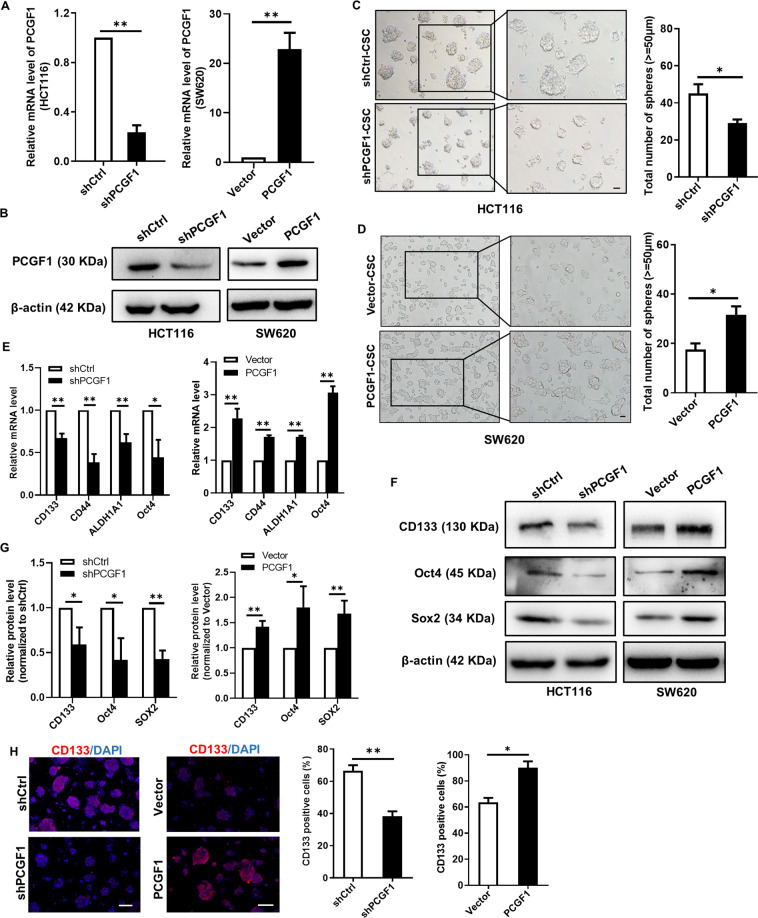
PCGF1 promotes colorectal cancer stem cell enrichment. **A**, **B** RT-qPCR and western blot analyses of PCGF1 in HCT116 cells silenced with PCGF1 shRNAs and RT-qPCR and western blot analysis of PCGF1 in SW620 cells that stably overexpress PCGF1. **C**, **D** Representative micrographs and quantification of tumour spheres formed by HCT116 and SW620 cells, and the number of spheres with diameters larger than 50 μm was counted. HCT116, Scale bar, 50 µm; SW620, Scale bar, 20 µm, right. **E** RT-qPCR analysis showed the mRNA expression levels of the stemness markers CD133, CD44, ALDH1A1 and Oct4 in CSC spheres with PCGF1 knockdown and overexpression. **F**, **G** Western blot analysis of stemness markers in PCGF1-overexpressing and PCGF1-silenced CSCs, and the relative intensity values (CD133/β-actin, Oct4/β-actin, Sox2/β-actin) were measured with ImageJ software. **H** Immunofluorescent staining of CD133 expression in CSCs. Bar graphs show the statistical data for CD133-positive cells. Scale bar, 100 µm. shCtrl, shControl. *p < 0.05. **p < 0.01, *t*-test. Data are shown as the mean ± SEM of at least three replicates. β-actin was used as a loading control.

A PCGF1 loss-of-function subclone was generated in HCT116 cells, which have high endogenous PCGF1 expression, using a pLKO-PCGF1 shRNA lentivirus, and a PCGF1 gain-of-function subclone was generated in SW620 cells, which have low endogenous PCGF1 expression, using a pLVX-PCGF1 lentivirus (Fig. 3A, B). PCGF1 knockdown strongly inhibited tumour sphere formation of HCT116 cells; the tumour spheres decreased in size, and the number of spheres (diameter ≥ 50 μm) notably decreased (Fig. 3C). Conversely, compared to vector control cells, SW620 cells overexpressing PCGF1 robustly promoted colorectal cancer stem cell sphere formation; the tumour spheres became larger and the number of spheres (diameter ≥ 50 μm) was increased (Fig. 3D). These results demonstrate that PCGF1 is essential for the maintenance of colorectal cancer stem cell properties and promotes colorectal cancer stem cell enrichment.

To gain insight into the mechanism by which PCGF1 promotes colorectal cancer stem cell enrichment, we investigated the relationships between PCGF1 and the stemness markers of colorectal cancer stem cells. Stemness markers reported for colorectal cancer stem cells include CD133, CD44, LGR5 and ALDH1A1 [4, 27]. The correlation between PCGF1 and the above stemness markers as follows: PCGF1 between CD133 (*R* = 0.17, *P* = 0.0027), CD44 (*R* = 0.38, *P* = 0.000), SOX2 (*R* = 0.19, *P* = 0.000), ALDH1A1 (*R* = 0.23, *P* = 0.000), LGR5 (*R* = 0.34, *P* = 0.000) and OCT4 (*R* = 0.52, *P* = 0.000) in primary CRC tissue samples in the GEPIA database (Supplementary Fig. 2). We further examined the regulatory effect of PCGF1 on the expression of stemness markers. RT-qPCR and western blot analyses showed that PCGF1 knockdown significantly reduced the expression of CD133, CD44, ALDH1A1, Sox2 and Oct4 in CRC stem cell spheres, which are enriched for CSCs. On the other hand, PCGF1 overexpression significantly increased the expression of stemness markers in CRC stem cell spheres (Fig. 3E, F and G). We also tested the effect of PCGF1 on CD133 expression by immunofluorescence staining and found that the number of CD133^+^ cells in the PCGF1-knockdown group was reduced and the number of CD133^+^ cells was increased in the PCGF1-overexpression group compared with the control group (Fig. 3H). These findings indicate that PCGF1 positively regulates the expression of colorectal cancer stemness markers.

### PCGF1 promotes colorectal cancer stem cell proliferation

**Fig. 4:**
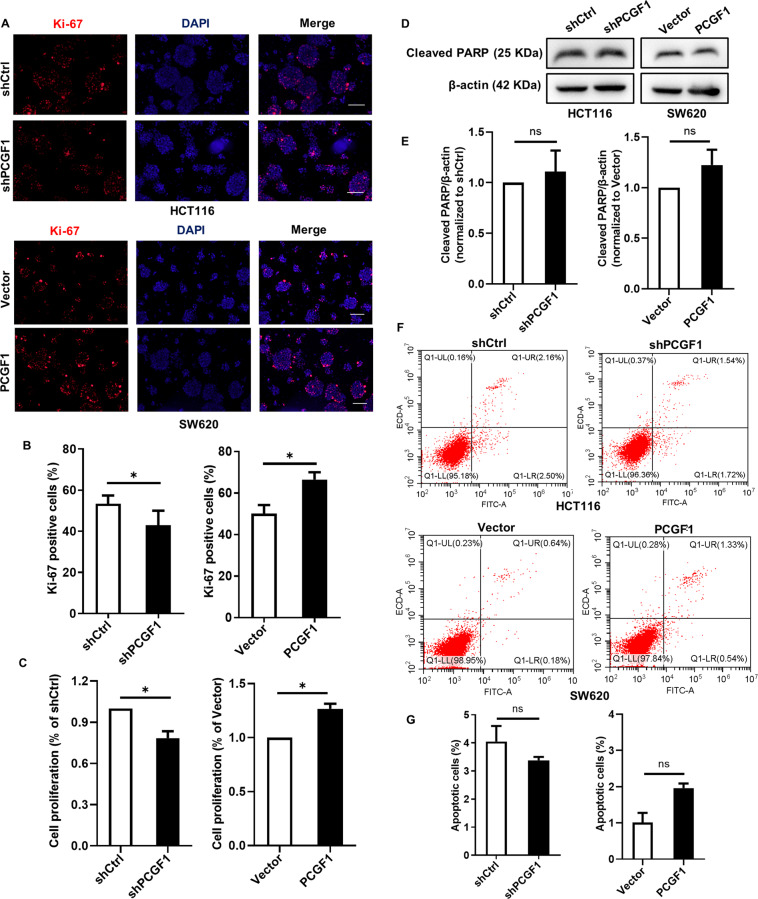
PCGF1 promotes colorectal cancer stem cell proliferation. **A**, **B** Proliferation was detected by Ki-67 staining. Bar graphs show the statistical data for the Ki-67-positive cells. Scale bar, 100 µm. **C** CRC stem cell proliferation was examined by a CCK-8 assay. **D**, **E** The expression of cleaved PARP was analysed by western blot, and the relative intensity values (cleaved PARP/β-actin) were measured with ImageJ software. **F**, **G** Cell apoptosis was examined by flow cytometry based on AV-PI staining. The histograms show the percentage of apoptotic cells. shCtrl, shControl. *p < 0.05*, t*-test. Data are shown as the mean ± SEM of at least three replicates. β-actin was used as a loading control.

We further tested the effect of PCGF1 on colorectal cancer stem cell proliferation using Ki-67 staining and a CCK-8 assay, and the results showed that cell proliferation was enhanced by PCGF1, while cell proliferation was reduced after PCGF1 knockdown (Fig. 4A–C). To verify the effect of PCGF1 on colorectal cancer stem cell apoptosis, the expression of the apoptosis-related protein cleaved PARP was determined using western blotting (Fig. 4D, E). AV-PI staining was used, and the number of apoptotic cells was determined using flow cytometry (Fig. 4F, G). The results showed that PCGF1 knockdown or overexpression did not change the apoptosis-related marker cleaved PARP or the number of apoptotic cells. Altogether, these results demonstrate that PCGF1 promoted colorectal cancer stem cell proliferation but had no significant effect on apoptosis.

### PCGF1 regulates colorectal cancer stem cell marker expression through histone modifications

**Fig. 5:**
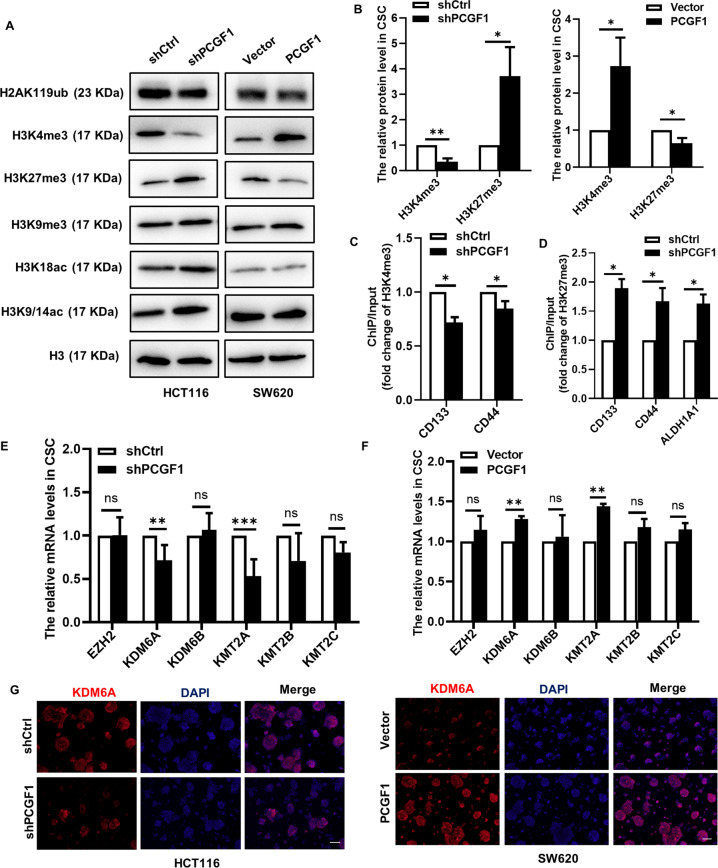
PCGF1 enhances colorectal cancer stemness marker transcription by increasing histone methylation at the promoters. **A**, **B** Western blot analysis of whole-cell lysates showed the expression of histone methylation and acetylation modification after PCGF1 silencing and overexpression, and the results were semiquantified using ImageJ software. **C**, **D** ChIP-qPCR analysis identified the expression of H3K4me3 and H3K27me3 at the promoters of CD133, CD44 and ALDH1A1 after PCGF1 knockdown in HCT116 cells. **E**, **F** The mRNA levels of the H3K27me3 methyltransferase EZH2; H3K27me3 demethylases KDM6A and KDM6B; and H3K4me3 methyltransferases KMT2A, KMT2B, and KMT2C in CSCs. **G** Immunofluorescent staining analysis of KDM6A expression in CSCs. Scale bar, 100 µm. shCtrl, shControl. ***P < 0.001, **P < 0.01, *P < 0.05*, t*-test. Data are shown as the mean ± SEM of at least three replicates. β-actin was used as a loading control.

PCGF1 is known as an important epigenetic regulator. Epigenetic modification of chromatin structure results in the activation or silencing of specific genes, which has been indicated to be an important molecular mechanism in cancer development [28]. In our study, we found that the expression levels of H3K4me3 were decreased while those of H3K27me3 were increased after PCGF1 knockdown. When PCGF1 was overexpressed, the expression levels of H3K4me3 were increased, while those of H3K27me3 were decreased. There were no significant changes in H2AK119ub, H3K9me3, H3K18ac or H3K9/K14ac (Fig. 5A, B). H3K4me3 marks transcriptional activation, while H3K27me3 marks transcriptional repression. To gain insight into the mechanism by which PCGF1 regulates colorectal cancer stem cell marker expression, ChIP-qPCR assays were used to determine whether PCGF1 regulates colorectal cancer stem cell marker expression through histone modifications. Interestingly, the results showed that the level of H3K4me3 was significantly decreased, while the level of H3K27me3 increased at the promoters of the stemness markers CD133, CD44 and ALDH1A1 after PCGF1 knockdown (Fig. 5C, D). These data imply that H3K4me3 and H3K27me3 modifications at the CD133, CD44 and ALDH1A1 promoters accounted for the PCGF1-mediated activation of these genes.

Next, we examined the histone modification machinery involved in H3K4me3 and H3K27me3 modifications using RT-qPCR. Our research found that PCGF1 affects the level of histone modification by regulating the expression of epigenetic modification enzymes, and the results showed that both the H3K27me3 demethylase KDM6A and the H3K4me3 methyltransferase KMT2A decreased significantly after PCGF1 knockdown, while the H3K27me3 methyltransferase EZH2, H3K27me3 demethylase KDM6B, and H3K4me3 methyltransferases KMT2B and KMT2C had no change (Fig. 5E). Both the expression levels of KDM6A and KMT2A were increased in PCGF1-overexpressing cells (Fig. 5F). Consistently, immunofluorescent staining showed that KDM6A increased in PCGF1-overexpressing cells but decreased in PCGF1-knockdown cells (Fig. 5G).

### PCGF1 knockdown inhibits tumour growth in vivo

**Fig. 6:**
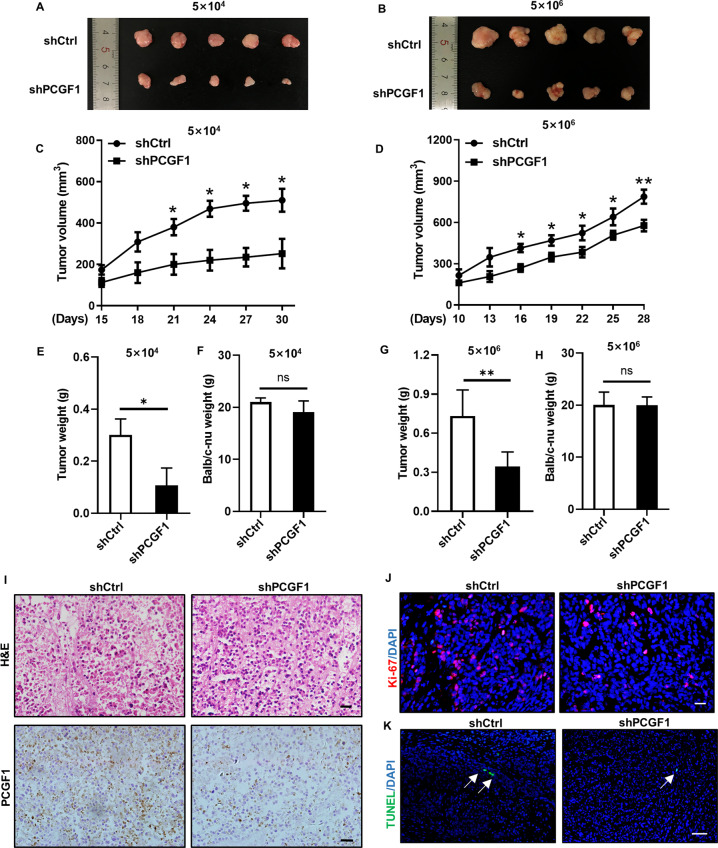
PCGF1 knockdown inhibits tumour growth in vivo. **A**, **B** Tumour size in nude mice after control and shPCGF1 HCT116 CRC cell injection on the day of harvest. **C**, **D** Tumour volume was measured every 3 days. Comparisons were performed for the shPCGF1 group versus the control group. **E**–**H** The histogram shows the average tumour weight and mouse weight of the two groups. *n* = 5 per group; error bars represent the SEM; **P < 0.01, *P < 0.05 versus control; one-way ANOVA and *t*-test. **I** Tumour sections were subjected to H&E staining and IHC using antibodies against PCGF1. H&E staining and IHC, scale bar, 20 µm. **J**, **K** Immunofluorescence staining of Ki-67 (red) and TUNEL (green) was performed to evaluate the proliferation of tumour cells. DAPI was used to stain the nuclei (blue). Ki-67 staining, scale bar, 20 µm; TUNEL staining, scale bar, 50 µm.

Prompted by the in vitro results, we further investigated the impact of PCGF1 in vivo. We inoculated control and PCGF1-knockdown HCT116 colorectal cancer cells (5 × 10^4^, 5 × 10^6^) subcutaneously into nude mice. Compared with the tumours from the control group, the tumours derived from PCGF1-knockdown HCT116 colorectal cancer cells were smaller (Fig. 6A, B). During the tumour-bearing period, tumour growth in the shPCGF1 group was significantly slower than that in control group, and the corresponding tumour volume was smaller (Fig. 6C, D). Then, the tumour weights of the two groups were analysed, and the results showed that the average weight of tumours in shPCGF1 group was significantly reduced compared to that in the control group (Fig. 6E, G). However, the body weights of the mice were not significantly altered (Fig. 6F, H). H&E and IHC were performed, and the IHC results confirmed that the expression of PCGF1 was suppressed by shPCGF1 (Fig. 6I). Tumour cell proliferation was determined by performing Ki-67 staining, and the results indicated that the tumour tissue derived from the shPCGF1 group displayed lower Ki-67-positive cells than that derived from the control group, demonstrating that PCGF1 inhibited the tumour cell proliferation in vivo (Fig. 6J). TUNEL staining was performed to assess tumour cell apoptosis, which indicated that there was no obvious difference between the two groups, demonstrating that PCGF1 has no significant effect on tumour cell apoptosis (Fig. 6K). Collectively, these results indicate that PCGF1 might be a promising therapeutic target that could be used to suppress CRC tumour growth in vivo.

## Discussion

CRC remains an incurable disease because 50% of patients with CRC experience tumour relapse and metastasis even if the tumour tissue is excised prior to tumour metastasis [29, 30]. Previous studies have identified that CSCs have intrinsic chemoresistant properties, ultimately resulting in chemotherapy failure and cancer recurrence [31]. Therefore, targeting CSCs should be a promising strategy for the treatment of colorectal cancer. Our study revealed that PCGF1 was overexpressed in CRC tissues and that PCGF1 expression was positively correlated with the expression of stemness markers, such as CD133 and CD44, in CRC tissues. Depletion of PCGF1 suppresses CRC stem cell proliferation and cancer stem cell enrichment. These observations suggest that PCGF1 is an important regulator of stemness and a potential diagnostic marker in CRC.

Recent studies have revealed that the PCGF family of proteins (PCGF1 (Nspc1), PCGF2 (Mel-18), PCGF3, PCGF4 (Bmi1), PCGF5 and PCGF6 is robustly elevated in diverse human cancers and promotes cancer progression [21, 32, 33]. However, the role of the PCGF family in CRC remains largely unknown. The GEPIA database predicts that only PCGF1 is significantly upregulated in both COAD and READ. We further predicted the expression of PCGF1 in various human cancers through the GEDS database and found that PCGF1 was generally more highly expressed in tumour tissue than in normal tissues. Studies also showed that PCGF1 could promote cell cycle progression and proliferation in HeLa and SH-SY5Y tumour cells [21]; PCGF1 is highly expressed in glioma cells and stem cell-like glioma cells (SLCs) and promotes the U87 glioma cell proliferation, colony formation [34] and self-renewal abilities of SLCs [22]. Herein, our results indicated that PCGF1 was markedly upregulated in colorectal cancer. PCGF1 expression positively correlated with tumour malignancy. In addition, higher PCGF1 expression tends to decrease the OS rate. Silencing of PCGF1 repressed CRC stem cell proliferation, enrichment of CSCs and tumour growth both in vitro and in vivo. Thus, our findings further suggest that PCGF1 plays a key role in the progression of CRC.

Solid tumours arise in organs that contain stem cell populations [35], and CSCs have been associated with the occurrence of colorectal cancer for a long time [36, 37]. PCGF proteins have been reported to be associated with the embryogenesis, self-renewal and differentiation of embryonic stem cells and are related to the biological activity of cancer stem cells [14, 38]. For instance, PCGF1 positively regulated the expression of essential transcription factors involved in ectoderm and mesoderm differentiation [18]. PCGF1 also promoted self-renewal of glioma stem cells by downregulating the expression of RDH16 [22]. PCGF4 has an essential role in regulating the proliferative activity of both normal and leukaemic stem cells [39]. PCGF6 directly regulates Oct4, Nanog, Sox2 and Lin28 expression to maintain ESC identity [40]. The above findings suggest that PCGF proteins may play vital roles in the regulation of stemness properties, including ESCs and CSCs. However, there are few reports on the effects of PCGF proteins on colorectal cancer stem cells. In this study, overexpression of PCGF1 enhanced colorectal cancer stem cell enrichment; conversely, silencing of PCGF1 inhibited tumour sphere formation. Moreover, PCGF1 epigenetically regulates the expression of the CSC markers CD133, CD44 and ALDH1A1, which are essential for maintaining CRC stemness properties. CD133^+^ human cancer cells were found to be capable of inducing CRC formation, while CD133^−^ cancer cells failed to do so [41]. In addition, CD133 expression was associated with poor prognosis [42, 43]. CD44 and ALDH1A1 are more specific CSC markers in CRC than CD133 [8]. Our results further show that PCGF1 played a vital role in maintaining the stemness of colorectal cancer stem cells.

As a member of the PcG family, PCGF1 was initially identified as a new mammalian polycomb gene that is highly expressed in the developing nervous system. Research on the function of PCGF1 mostly focuses on stem cell self-renewal and embryonic development [18, 20]. With the in-depth study of PCGF1, the role of PCGF1 in tumour occurrence and development has been gradually revealed [22, 34]. Canonically, PCGF1 is characterized as a transcriptional repressor that interacts with KDM2B, RING1B and other components to jointly regulate gene transcription and expression [44]. As an epigenetic regulator, PCGF1 has been shown to inhibit gene transcription through H2AK119ub1 and H3K27me3. Previously, PCGF1 was shown to enhance H2AK119ub1, and siRNA-mediated knockdown of PCGF1 reduced the H2A ubiquitination levels in HeLa cells [45]. PRC1 caused H2AK119ub1 through RING1A/B and could promote the enrichment of downstream PRC2 and H3K27me3 [46]. The noncanonical roles of PCGF1 in transcriptional regulation have also been documented. Hui Li et al. [47] reported that PCGF1 activated Oct4 by directly binding to the (−1021 to −784) region of the Oct4 promoter in P19 embryonal carcinoma cells. These studies suggest that the exact roles of PCGF1 in transcriptional regulation remain largely undefined. In our study, we showed that PCGF1 binds to promoters of the CRC stem cell markers CD133 and CD44 and activates their transcription by increasing H3K4me3 and decreasing H3K27me3 marks on the promoters, which is consistent with previous studies showing that PCGF1 is involved in epigenetic regulation of gene expression. Our study further enriched the research on the regulatory role of PCGF1 in CSCs.

PCGF1 belongs to the PcG protein family, which contains epigenetic regulators of transcription that have key roles in stem cell identity, differentiation and disease [14]. Epigenetic modifications are closely correlated with gene transcription. H3K27me3, which is always bound by PRC1 complexes, marks transcriptional repression, while H3K4me3 marks transcriptional activation [48, 49]. Our data indicate that loss of PCGF1 decreases H3K4me3 and increases H3K27me3 at the promoters of stemness markers, suggesting that PCGF1 activates the stemness markers CD133 and CD44 through the H3K4me3 and H3K27me3 modifications in the promoter region of colorectal cancer stem cells. Moreover, during DNA transcription, methyltransferases and demethylases are involved in histone modification. The H3K27me3 demethylase KDM6A; methyltransferase EZH2; H3K4me3 methyltransferases KMT2A, KMT2B and KMT2C; and H3K4me3 demethylase LSD1 get involved in H3K4me3 and H3K27me3 modifications [50]. In our study, we discovered that PCGF1 upregulated the expression of stemness markers by maintaining the histone H3K4me3 status by increasing the expression of KMT2A and removing the histone H3K27me3 status by increasing the expression of KDM6A, suggesting that KDM6A and KMT2A play a vital role in colorectal cancer stem cells.

**Fig. 7:**
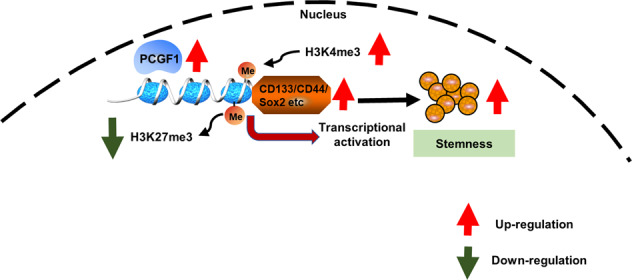
A diagram of the mechanisms by which PCGF1 promotes colorectal cancer stem cell enrichment. Schematic figure showing that PCGF1 promotes colorectal cancer stem cell enrichment by maintaining H3K4me3 marks and removing H3K27me3 marks at the promoters of stemness markers.

In summary, we have demonstrated that PCGF1 promotes colorectal cancer stem cell enrichment and cell proliferation in vivo and in vitro. Mechanistically, PCGF1 promotes the expression of the colorectal cancer stemness markers CD133, CD44 and ALDH1A1 by maintaining histone H3K4me3 and removing histone H3K27me3 marks by increasing the expression of the H3K4me3 methyltransferase KMT2A and the H3K27me3 demethylase KDM6A. This study provides potential therapeutic targets for reversing stemness and enhancing chemosensitivity in CRC (Fig. 7).

## Supplementary information

SUPPLEMENTAL MATERIAL

## Data Availability

The datasets generated and/or analysed during the current study are included within the article and are available from the corresponding authors on reasonable request.
