# Supplementary material for: PCGF1 promotes epigenetic activation of stemness markers and colorectal cancer stem cell enrichment
Source: Cell Death Dis. 2021 Jun 19;12(7):633. doi: 10.1038/s41419-021-03914-2 (PMC8214626; doi:10.1038/s41419-021-03914-2)
Supplement: Supplementary file 1 — SUPPLEMENTAL MATERIAL [file 41419_2021_3914_MOESM1_ESM.docx]

**Supplementary Materials**


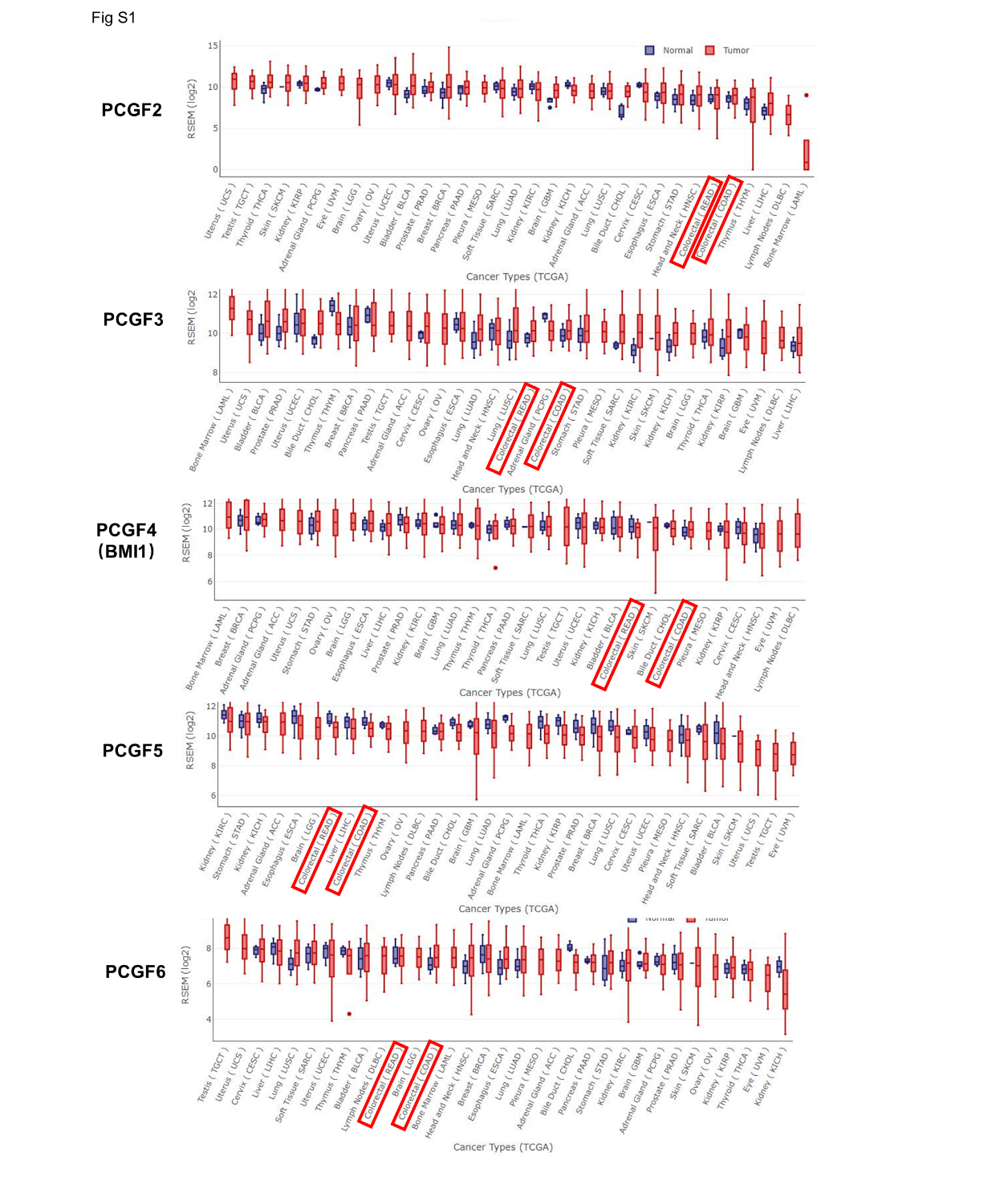


Fig. S1 The expression of PCGF2-6 in various types of tumours using the GEDS database. The red boxes refer to colorectal READ and COAD.


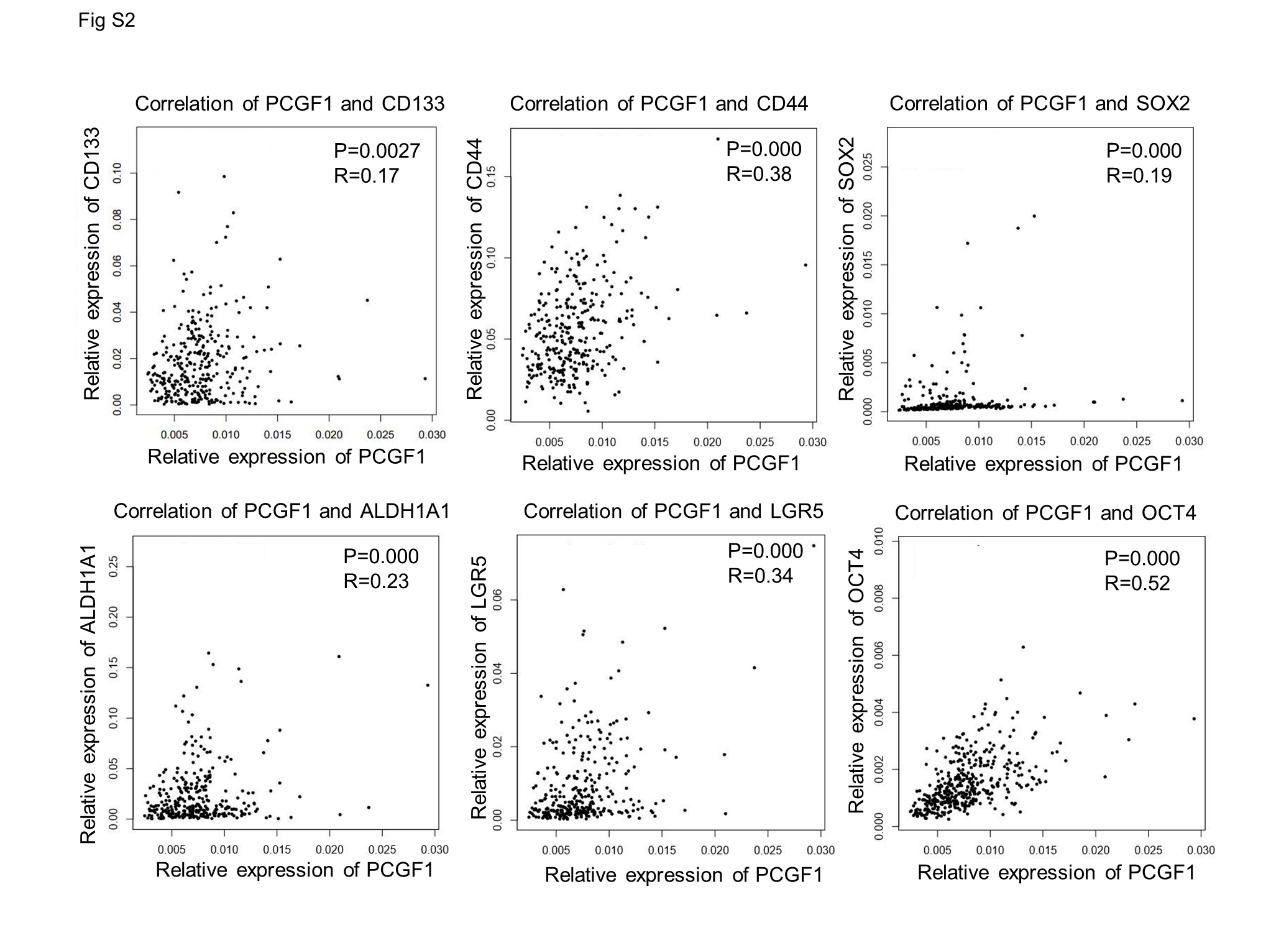


Fig. S2 PCGF1 is positively correlated with colorectal cancer stemness markers. The graph shows the relationship of PCGF1 expression and the expression of the stem cell markers CD133, CD44, LGR5, ALDH1A1, SOX2 and OCT4 in the GEPIA database.
